# Current‐Induced Reversible Split of Elliptically Distorted Skyrmions in Geometrically Confined Fe_3_Sn_2_ Nanotrack

**DOI:** 10.1002/advs.202206106

**Published:** 2023-01-22

**Authors:** Zhipeng Hou, Qingping Wang, Qiang Zhang, Senfu Zhang, Chenhui Zhang, Guofu Zhou, Xingsen Gao, Guoping Zhao, Xixiang Zhang, Wenhong Wang, Junming Liu

**Affiliations:** ^1^ Guangdong Provincial Key Laboratory of Optical Information Materials and Technology & Institute for Advanced Materials South China Academy of Advanced Optoelectronics South China Normal University Guangzhou 510006 P. R. China; ^2^ College of Electronic information and automation Aba Teachers University Pixian Street Chengdu 623002 China; ^3^ College of Physics and Electronic Engineering Sichuan Normal University Chengdu 610068 China; ^4^ Core Technology Platforms New York University Abu Dhabi P.O. Box 129188 Abu Dhabi United Arab Emirates; ^5^ Physical Science and Engineering Division King Abdullah University of Science and Technology Thuwal 23955‐6900 Saudi Arabia; ^6^ School of Electronic and Information Engineering Tiangong University Tianjin 300387 China; ^7^ Laboratory of Solid State Microstructures and Innovation Center of Advanced Microstructures Nanjing University Nanjing 211102 China

**Keywords:** current‐driven dynamics, room temperature, skyrmions

## Abstract

Skyrmions are swirling spin textures with topological characters promising for future spintronic applications. Skyrmionic devices typically rely on the electrical manipulation of skyrmions with a circular shape. However, manipulating elliptically distorted skyrmions can lead to numerous exotic magneto‐electrical functions distinct from those of conventional circular skyrmions, significantly broadening the capability to design innovative spintronic devices. Despite the promising potential, its experimental realization so far remains elusive. In this study, the current‐driven dynamics of the elliptically distorted skyrmions in geometrically confined magnet Fe_3_Sn_2_ is experimentally explored. This study finds that the elliptical skyrmions can reversibly split into smaller‐sized circular skyrmions at a current density of 3.8 × 10^10^ A m^−2^ with the current injected along their minor axis. Combined experiments with micromagnetic simulations reveal that this dynamic behavior originates from a delicate interplay of the spin‐transfer torque, geometrical confinement, and pinning effect, and strongly depends on the ratio of the major axis to the minor axis of the elliptical skyrmions. The results indicate that the morphology is a new degree of freedom for manipulating the current‐driven dynamics of skyrmions, providing a compelling route for the future development of spintronic devices.

## Graphical abstract

Current‐driven split and integration of the elliptically distorted skyrmions are realized. At a current density of 3.8 × 10^10^ A m^−2^, the elliptical skyrmions split into two circular skyrmions. More importantly, such a transition is reversible, which is beneficial for memory applications. The results establish a viable approach to manipulating skyrmions, significantly broadening the design of skyrmion‐based devices.

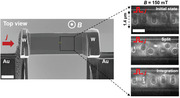

## 1 Introduction

Magnetic skyrmions, which are swirling spin configurations with quantized topological charge, have attracted tremendous attention over the past decade due to their promising application for future high‐density, low‐dissipation spintronic devices. Hitherto, skyrmions are generally observed in non‐centrosymmetric B20 compounds^[^ ^1^, ^2^, ^3^, ^4^, ^5^, ^6^, ^7^, ^8^, ^9^, ^10^ ^]^ as well as asymmetric multilayer films.^[^ ^11^, ^12^, ^13^, ^14^, ^15^, ^16^, ^17^ ^]^ In these materials, broken inversion symmetry generates a Dzyaloshinskii–Moriya interaction (DMI), which not only stabilizes skyrmions but also endows them with a fixed chirality. In addition to the DMI‐hosting magnetic systems, research communities have demonstrated that centrosymmetric ferromagnets could stabilize skyrmions (or called skyrmionic bubbles and chiral skyrmions) even without significant DMI, and the interplay of magnetic exchange interaction, uniaxial anisotropy, and dipole‐dipole interaction plays a dominant role in their formation.^[^ ^18^, ^19^, ^20^, ^21^, ^22^, ^23^, ^24^, ^25^, ^26^, ^27^, ^28^, ^29^ ^]^ Due to the absence of DMI, the helicity and vorticity of skyrmions in centrosymmetric ferromagnets possess two additional degrees of freedom compared with those of DMI‐stabilized skyrmions. However, from the topological point of view, the two types of spin configurations are approximately equivalent.^[^ ^18^, ^19^, ^20^, ^21^, ^22^, ^23^, ^24^, ^25^, ^26^, ^27^, ^28^, ^29^ ^]^ As a consequence, they display similar magneto‐electronic functions, including the skyrmion Hall effect,^[^ ^11^, ^24^, ^27^ ^]^ the topological Hall effect,^[^ ^10^, ^19^ ^]^ and the ultralow threshold current for motion.^[^ ^26^, ^30^ ^]^

**Figure 1:**
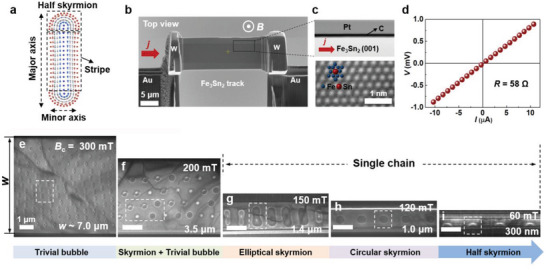
Schematic and magnetic domain states of track devices with different widths. a) Schematic of an elliptical skyrmion. The component domains are enclosed by the black dashed boxes. b) Top view of the Fe_3_Sn_2_ nanotrack device. The red arrow represents the injected current. The scale bar is 5 µm. c) Upper panel shows the STEM image of the track enclosed by the black box in (b). Lower panel shows the HR‐STEM image taken along the normal direction of the track. Blue and red particles represent the Fe and Sn atoms, respectively. d) *I*–*V* curve of the track device. e–i) LTEM images taken on the tracks with different widths under their corresponding critical field. The typical magnetic domain states in each image are enclosed by the white dashed boxes. The scale bars in e‐i represent 1 µm.

For spintronic applications, skyrmions must be controllably manipulated via electrical means in defined geometries.^[^ ^1^, ^2^, ^3^, ^4^ ^]^ To date, these operations are generally implemented on the basis of skyrmions with a circular shape.^[^ ^1^, ^2^, ^3^, ^4^ ^]^ In this scenario, their dynamic behaviors are isotropic due to the circular symmetry. However, the spatially confined effect of the geometries can break the circular symmetry and force the skyrmions to be elliptically distorted in morphology without changing their topological class.^[^ ^31^, ^32^ ^]^ An elliptically distorted skyrmion can be regarded as the combination of a stripe domain (topological charge is “0”) and two half‐circular skyrmions attached at the end of the stripe (topological charge of a half skyrmion is “1/2” and two half skyrmions form a skyrmion),^[^ ^33^, ^34^ ^]^ as schematically illustrated in **Figure** **1**a. Because the topological charge is strongly coupled with the current, the dynamic behaviors of the half skyrmions are strikingly different from those of the stripe domain with respect to the electrical stimuli.^[^ ^33^, ^34^ ^]^ Theoretical simulations have demonstrated that such inhomogeneous current responses of the component domains could force the integral elliptical skyrmion to be stretched and split into smaller‐sized skyrmions.^[^ ^33^ ^]^ This exotic magneto‐electrical property is distinct from that of the conventional circular skyrmions and can lead to the variation of the number of skyrmions in a defined geometry, holding promise for applications beyond conventional binary memories, such as multilevel memories^[^ ^1^, ^35^, ^36^ ^]^ and neuromorphic computing.^[^ ^35^, ^36^, ^37^ ^]^ Nevertheless, despite the promising potential, experimental realization of the current‐driven split of the elliptical skyrmions so far remains elusive.

To implement the skyrmion split, a crucial step is to create elliptically distorted skyrmions. Previous investigations have demonstrated that geometrically confined skyrmions in FeGe nanotrack could adopt a wide range of ellipticities.^[^ ^31^, ^32^ ^]^ However, their stable temperature is far below room temperature. By contrast, the frustrated magnet Fe_3_Sn_2_, which possesses a centrosymmetric rhombohedral crystal structure, is known to be able to host skyrmions over a wide range of temperatures from 100 K up to 630 K (the Curie temperature *T*_c_ of Fe_3_Sn_2_ is ≈640 K).^[^ ^18^, ^29^, ^38^, ^39^ ^]^ Such high thermal stability makes Fe_3_Sn_2_ a suitable material platform for both fundamental research and spintronic applications. In this work, we targeted the centrosymmetric frustrated magnet Fe_3_Sn_2_ and systematically studied the current‐driven dynamics of the elliptically distorted skyrmions in the geometrically confined Fe_3_Sn_2_ nanotrack using in situ Lorenz transmission electron microscopy (LTEM). We found that the elliptical skyrmions could split into smaller‐sized circular skyrmions at a current density of 3.8 × 10^10^ A m^−2^ with the current injected along their minor axis. More importantly, such a transition is reversible, which is crucial for memory applications. Combined experiments and micromagnetic simulations revealed that these dynamic behaviors originate from a delicate interplay of the spin‐transfer torque (STT), geometrically confined effect, and pinning effect, and strongly depend on the ratio of the major axis to the minor axis of the elliptical skyrmions. Our results suggest that the morphology can be considered a new degree of freedom to manipulate the dynamics of skyrmions, significantly broadening the scope of means in the design of innovative spintronic devices.

## 2 Results and Discussion

### 2.1 Fabrication of Fe_3_Sn_2_ Track Devices

First, we fabricated the track devices that allow us to directly explore the current‐driven behaviors of skyrmions in LTEM, from a Fe_3_Sn_2_ single crystal using the focused ion beam (FIB) technique. Figure 1b depicts the detailed structure of a typical track device, in which a [001]‐oriented Fe_3_Sn_2_ nanotrack is fixed on a pair of gold (Au) electrodes through the deposition of tungsten (W) on their joints. The surrounding parts of the nanotrack are coated with carbon (C) and platinum (Pt) to suppress the Fresnel fringes in the LTEM images (see the upper panel of Figure 1c). A high‐resolution scanning transmission electron microscopy (HR‐STEM) image taken along the normal direction of the Fe_3_Sn_2_ nanotrack reveals that Fe and Sn atoms are orderly arranged into the kagome lattices (see the lower panel of Figure 1c), confirming the [001]‐orientation. Figure 1d illustrates the current–voltage (*I*–*V*) curve of the device. *I* exhibits a linear dependence on *V*, revealing that the device has an ohmic contact. Moreover, the corresponding resistance (*R*) is as low as 58 Ω, suggesting the high quality of the device.

Subsequently, we explored the magnetization dynamics of the track devices with different widths (*w*) to establish the *w* range for hosting the elliptically distorted skyrmions. The magnetic field (*B*) was applied along the out‐of‐plane direction of these devices and the *w* was designed to range from 4.9 µm to 300 nm with a fixed thickness (*t*) of ≈250 nm (see Figure S1, Supporting Information). Figure 1e–i presents *w*‐dependent LTEM images under their corresponding critical magnetic field (*B*_c_), at which spontaneous stripe domains completely transform into bubble domains. As illustrated in Figure 1e, the *w* ≈ 4.9 µm track hosted trivial bubbles at *B*_c_ = 300 mT. With decreasing *w*, the skyrmions gradually isolated from the trivial bubbles due to the strengthening of the geometrically confined effect (see Figure 1f–i).^[^ ^39^ ^]^ In particular, when *w* fell below 1.4 µm (including 1.4 µm), the trivial bubbles were completely excluded and only the skyrmions existed in the nanotrack. Remarkably, these skyrmions were arranged densely into single chains, and their morphology underwent a continuous transition from an ellipse to a circle, and then to a half‐circle with decreasing *w* (see Figure 1g–i). The *w*‐dependent morphological variation demonstrates a flexible feature of the skyrmions^[^ ^31^, ^32^ ^]^ and is expected to influence their current‐driven dynamics significantly. Moreover, we found that some of the skyrmions exhibited a completely inverse contrast variation in the LTEM image, as denoted in Figure 1g. Combined experiments and micromagnetic simulations revealed that such an obvious contrast variation was because the swirling direction of these skyrmions’ in‐plane spin was inversed; namely, their helicity was inversed (see Figure S2, Supporting Information). Notably, the unfixed helicity is common for the skyrmions in centrosymmetric magnets^[^ ^18^, ^19^, ^20^, ^21^, ^22^, ^23^, ^24^, ^25^, ^26^, ^27^, ^28^, ^29^ ^]^ and can be attributed to the absence of DMI.

### 2.2 Current‐Driven Dynamics of Elliptically Distorted Skyrmions

**Figure 2:**
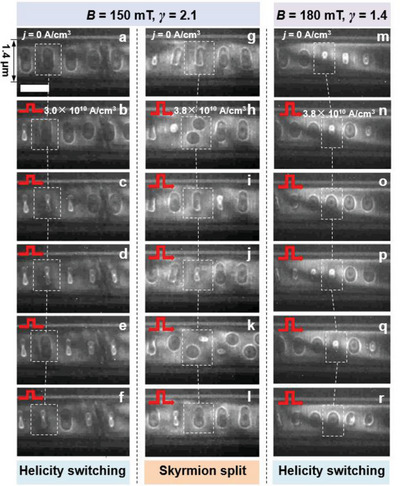
Current‐driven dynamics of the elliptical skyrmions in the *w* ≈ 1.4 µm track device. a,g) LTEM images of the *γ* = 2.1 elliptical skyrmions at *j* = 0 A m^−2^ and an out‐of‐plane magnetic field (*B*) of 150 mT. LTEM images after injecting a series of current pulses with a density of b–f) 3.0 × 10^10^ A m^−2^ and h–l) 3.8 × 10^10^ A m^−2^. m) LTEM images of the *γ* = 1.4 elliptical skyrmions at *j* = 0 A m^−2^ and *B* = 180 mT. LTEM images after injecting a series of current pulses with a density of n–r) 3.8 × 10^10^ A m^−2^. The magnetic states enclosed by white dashed boxes show one‐to‐one correspondence. The red arrow points to the direction of the injected current flow. The scale bar is 1 µm.

Having established that the *w* ≈ 1.4 µm track device hosts elliptically distorted skyrmions (see Figure 1g), we then explored their current‐driven dynamics. The current pulses were injected along the minor axis of the elliptical skyrmions from the Au electrodes (see Figure 1b), and the pulse width (*τ*) and frequency (*f*) were fixed at 100 ns and 1 Hz, respectively. The density (*j*) of the current pulses ranged from 0 to a maximum value of 4.2 × 10^10^ A m^−2^, above which it is easy to heat the track devices above *T*_c_ of Fe_3_Sn_2_.^[^ ^29^ ^]^ The current‐driven dynamics were recorded as LTEM movies with a frame rate of 60 frames per second, and these movies were analyzed to evaluate the change in magnetization configurations. For *j* < 3.0 × 10^10^ A m^−2^ and *B* = 150 mT, no directional motion or structural variation was observed in the elliptical skyrmions. However, once *j* was increased above 3 × 10^10^ A m^−2^ (≥3.0 × 10^10^ A m^−2^), their spin textures began to vary significantly. Movies S1 and S2 (Supporting Information) record the dynamic behaviors at two critical *j* values (i.e., *j* = 3.0 × 10^10^ and 3.8 × 10^10^ A m^−2^), at which the current responses of the elliptical skyrmions are strikingly different. To illustrate the details of the variations, we extracted a series of sequential LTEM images from these movies, and the corresponding images are presented in **Figure** **2**a–l. The skyrmions enclosed by the white boxes represent the one‐to‐one correspondence. As shown in Figure 2a–f, at a relatively lower *j* of 3.0 × 10^10^A m^−2^, helicity‐switching was observed in the elliptical skyrmions. Notably, this dynamic behavior has also been observed in the circular configurations (see Movie S3, Supporting Information and our previous reports^[^ ^29^ ^]^) indicating that the helicity variation is independent of the skyrmion morphology. However, when *j* was increased up to 3.8 × 10^10^ A m^−2^, the dynamic behaviors varied strikingly and it was interesting to find that some of the elliptical skyrmions split into circular skyrmions with smaller sizes (see Figure 2g–l). Due to the geometrically confined effect, these circular skyrmions were orderly arranged into a zigzag chain along the longitudinal direction of the nanotrack (see Figure 2h,k). By further injecting the current pulse, the smaller skyrmions could be reversely integrated into the elliptical skyrmions, regardless of whether their helicity was the same or the opposite (see Figure 2h–l). Notably, the split‐integration transition did not occur after certain current pulses (see Figure 2i,j), suggesting a local energy fluctuation in the track device.^[^ ^40^ ^]^ Moreover, we discovered that, within the *j* range from 3.0 × 10^10^ to 3.8 × 10^10^ A m^−2^, the current pulse was unable to drive a continuous, directional skyrmion motion (see Movies S1–S3, Supporting Information), which has been widely reported in previous theoretical and experimental studies.^[^ ^11^, ^12^, ^13^, ^27^, ^41^ ^]^ The absence of skyrmion motion could be attributed to the dense arrangement of skyrmions along the nanotrack, which led to a strong pinning effect on the skyrmion motion due to the repulsive potential between neighboring skyrmions.^[^ ^42^, ^43^ ^]^ Furthermore, we explored the dynamic behaviors of the elliptical skyrmions at a higher *B* of 180 mT. As shown in Figure 2m, the increase in *B* reduces the major axis of the elliptical skyrmions, which makes the length ratio (*γ*) of the major axis to the minor axis decrease from an average value of 2.1 at *B* = 150 mT (see Figure 2a) to 1.4 at *B* = 180 mT (see Figure 2m). However, the skyrmion split could no longer be observed after a series of current pulses with *j* = 3.8 × 10^10^ A m^−2^ (see Figure 2m–r), suggesting that the dynamic behavior depends on the value of *γ*. A detailed discussion of the relationship between the skyrmion split and *γ* is presented below.

## 3 Discussion

The aforementioned experiments revealed a series of current‐driven dynamic behaviors of the elliptical skyrmions on the basis of the Fe_3_Sn_2_ track devices. Next, we discuss the underlying physical mechanism. As is well known, the injected pulse current generates not only the STT effect but also Joule heating,^[^ ^44^, ^45^ ^]^ both of which may significantly affect the dynamics of skyrmions. To rule out the Joule heating effect, we performed simulations to study the relationship between Joule heating and current density. Details about the simulations are shown in Note S1 (Supporting Information). We first simulated the temperature distribution of the nanotrack at *j* = 3.0× 10^10^ and 3.8 × 10^10^ A m^−2^, where a current‐induced helicity switching (*j* = 3.0 × 10^10^) and skyrmion split (*j* = 3.8 × 10^10^ A m^−2^) was observed, respectively. It is found that the highest temperature of the nanotrack at *j* = 3.0× 10^10^ and 3.8 × 10^10^ A m^−2^ is 430 and 550 K, respectively (see Figure S3, Supporting Information). Furthermore, we studied the thermal stability of the elliptic skyrmions at the temperature range of 300–550 K (see Figure S4, Supporting Information). It is clearly demonstrated that both the helicity and structure of the elliptic skyrmions are very stable in the absence of the pulse current. Thus, we can conclude that the observed helicity switching and split of the elliptic skyrmions are not directly triggered by the Joule heating but by the STT effect. However, although Joule heating is not the dominant factor for the current‐driven behaviors, the thermal effect is proposed to be beneficial for the switching process, because the thermally activated energy is demonstrated to be able to decrease the energy barrier in the dynamic behaviors.^[^ ^44^ ^]^

**Figure 3:**
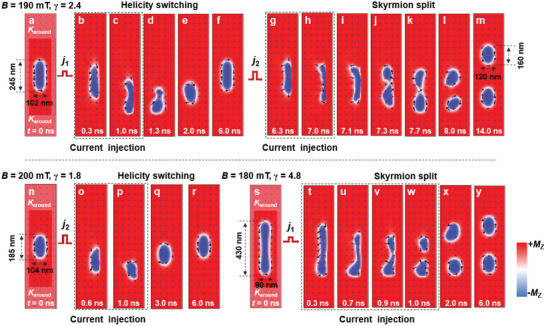
Simulated current‐driven dynamics of elliptical skyrmions. a) Snapshots of an elliptical skyrmion with *γ* = 2.4 at *t* = 0 ns (without injection of current). The length, width, and thickness of the track are 1 µm, 250 nm, and 100 nm, respectively. The shadow region represents the pining region with a pinning strength of *K*_around_ = 20*K*_u_. b–f) Snapshots at five selected times showing the helicity switching of the elliptical skyrmion. A spin‐polarized current of 2.0 × 10^12^ A m^−2^ is injected during *t* = 0–1 ns. g–m) Snapshots at seven selected times showing the split of the elliptical skyrmion at a current of 3.0 × 10^12^ A m^−2^. The current is injected during *t* = 6–7 ns. An out‐of‐plane magnetic field (*B*) of 190 mT is applied for (a)–(m). n) Snapshots of an elliptical skyrmion with *γ* = 1.8 at *t* = 0 ns. o–r) Snapshots at four selected times showing the helicity switching at a current of 3.0 × 10^12^ A m^−2^. The value of *B* for (n)–(r) is 200 mT. s) Snapshots of an elongated skyrmion with *γ* = 4.8 at *t* = 0 ns. t–y) Snapshots at six selected times showing the helicity switching at a current of 2.0 × 10^12^ A m^−2^. The value of *B* for (s)–(y) is 180 mT. The magnetization along the *z*‐axis (*M*_z_) is represented by regions in red (+*M*_z_) and blue (−*M*_z_).

To gain a deeper insight into the role of the STT effect, we explored the current‐driven behaviors of the elliptical skyrmions using micromagnetic simulations. First, the magnetization process of the elliptical skyrmions was simulated considering the interplay of ferromagnetic exchange interaction, uniaxial anisotropy, and dipole–dipole interaction (see the Experimental Section). A continuous evolution was realized from the stripe domain to an elliptical skyrmion (see Figure S5, Supporting Information). The simulated evolution process agreed well with the experimental results, thus validating our numerical model. Subsequently, the current‐driven dynamic behaviors of the elliptical skyrmions were simulated considering the adiabatic and non‐adiabatic STT effects on the basis of the Zhang‐Li model,^[^ ^46^ ^]^ in which the spins of the conduction electrons are polarized by the local magnetic moments and follow their magnetization direction. Moreover, because the skyrmions were strongly pinned in the track device, we set high magnetocrystalline anisotropy (*K*_u_) regions with a pinning strength (*K*_around_) of 20*K*_u_ around the simulated skyrmion to prevent their transverse motion (see the shadow regions in **Figure** **3**a). A detailed description of the simulations was presented in the Experimental Section. Figure 3a–m depicts a series of current‐driven dynamic snapshots of an elliptical skyrmion with a length ratio (*γ*) of 2.4 (the lengths of the major and minor axes are 245 and 102 nm, respectively). When a relatively weaker current of *j*_1_ was injected, the helicity was reversed, accompanied with the destruction of the skyrmionic structure (see Figure 3a–f). However, when the current density was increased to a larger value of *j*_2_, the helicity‐switching could no longer be observed; instead, the elliptical skyrmion split into two skyrmions with smaller *γ* (see Figure 3g–m). This result suggests that helicity‐switching is a more favorable option than the split under electric excitation. By further injecting the current of *j*_2_, the smaller skyrmions were reversely integrated into an elliptical skyrmion, and then the restructured elliptical skyrmion split into two smaller‐sized skyrmions (see Figure S6, Supporting Information). These simulated results are consistent with the experimental observations (see Figure 2a–l), which not only confirms that our theoretical model is reliable but also reveals that the STT effect dominates the splitting behavior.

We further simulated the current‐driven dynamics of the elliptical skyrmions with different *γ* values. As displayed in Figure 3n–r, when *γ* was decreased from 2.4 to 1.8 (the lengths of the major and minor axes are 185 and 104 nm, respectively) by increasing *B*, the split disappeared, and only helicity‐switching could be observed until the skyrmion was annihilated. However, when *γ* was increased to 4.8 (the lengths of the major and minor axes are 430 and 90 nm, respectively), only the split was triggered (see Figure 3s–y). These simulations demonstrated that the dynamic behaviors of the elliptical skyrmions are closely related to the value of *γ*. As mentioned earlier, an elliptical skyrmion can be regarded as the combination of a stripe domain and two half‐skyrmions or a circular skyrmion, as schematically illustrated in Figure 1a. When a spin‐polarized current is injected into the strongly pinned elliptical skyrmion, the STT effect could lead to two independent behaviors; specifically, the STT effect tended to induce helicity‐switching in the circular skyrmion and squash the stripe domain along the current direction (see Figure S7, Supporting Information). We propose that the competition between the two types of STT effect leads to the *γ*‐dependent dynamics. Regarding the *γ* = 1.8 skyrmion, because its major axis was only slightly elongated (i.e., the stripe domain accounted for a small proportion of the entire skyrmion), the helicity‐switching induced by the STT effect dominated the squashing effect during the evolution process. Thus, only helicity switching was observed (see Figure 3n–r). As the proportion of the stripe domain increased, the squashing effect became increasingly pronounced. When this effect became dominant, the minor axis of the elliptical skyrmion decreased correspondingly with respect to the current (see Figure 3g–j and s–v). Once the domain walls on the left and right sides were connected, the elliptical skyrmion would split into two smaller ones, as demonstrated in Figure 3k–m and v–y.

**Figure 4:**
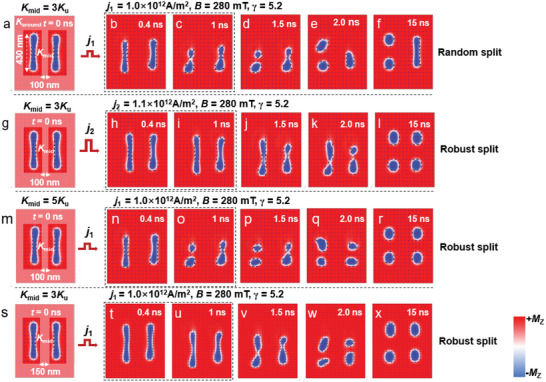
Simulated current‐driven dynamics of an elliptical skyrmion chain. a–f) Snapshots showing the dynamics of the elliptical skyrmions at *B* = 280 mT and *γ* = 5.2. A spin‐polarized current of 1.0 × 10^12^ A m^−2^ is injected during *t* = 0–1 ns. The length, width, and thickness of the track is 1 µm, 800 nm, and 100 nm, respectively. The shadow region represents the pining region with a pinning strength of *K*_around_ = 20*K*_u_, *K*_mid_ = 3*K*_u_. The width (*w*_mid_) of *K*_mid_ is 100 nm. g–l) Snapshots showing the dynamics at *j =* 1.1 × 10^12^ A m^−2^ (the other parameters are the same as those of (a)–(f). m–r) Snapshots showing the dynamics for *K*_mid_ = 5*K*_u_ (the other parameters are the same as those of (a)–(f). s–x) Snapshots showing the dynamics for *w*_mid_ = 150 nm (the other parameters are the same as those of (a)–(f)). The magnetization along the *z*‐axis (*M*_z_) is represented by regions in red (+*M*_z_) and blue (−*M*_z_).

Furthermore, our experiments demonstrated that the split behavior occurred only in parts of the skyrmions even at the maximum critical current density of 4.2 × 10^10^ A m^−2^. To understand the physical origin of the random feature, we simulated the split process of an elliptical skyrmion chain including two *γ* = 5.2 elliptical skyrmions (see **Figure** **4**). It was found that the split of the skyrmion chain was closely related to the value of *j* injected (see Figure 4a–l); specifically, the split occurred in only one skyrmion at a relatively smaller *j*_1_ (see Figure 4a–f), whereas it occurred in both of the skyrmions when a relatively larger *j*_2_ was injected (see Figure 4g–l). Moreover, we discovered that, if the strength of the pinning region between the two elliptical skyrmions (*K*_mid_) increased from 3*K*_u_ to 5*K*_u_ or the width (*w*_mid_) of the pinning region between two elliptical skyrmions increased from 100 to 150 nm, the split occurred in both of the skyrmions at a relatively smaller *j*_1_ (see Figure 4m–x). We thus propose that the random split observed in our experiments might be attributed to the following two reasons: i) the maximum critical density of the injected current (*j* = 4.2 × 10^10^ A m^−2^) was still below the threshold required for inducing a uniform split; ii) the distribution of *K*_mid_ and *w*_mid_ was inhomogeneous and the values of *K*_mid_ and *w*_mid_ in certain regions were insufficient to generate a robust split.

## 4 Conclusions

In summary, we have systematically studied the current‐driven behaviors of the elliptically distorted skyrmions with the current injected along their minor axis in the geometrically confined Fe_3_Sn_2_ track devices using in situ LTEM. We found that some of the elliptical skyrmions can split into circular skyrmions of smaller sizes at a current density of 3.8 × 10^10^ A m^−2^. Moreover, the split is reversible, which is highly useful for constructing spintronic devices. Combined with the micromagnetic simulations, our results demonstrated that the observed dynamic behaviors originate from a delicate interplay of STT, geometrically confined effect, and pinning effect, and strongly depend on the length ratio of the major axis to the minor axis of the elliptical skyrmions. These results indicate that morphology can be considered a degree of freedom for manipulating the current‐driven dynamics of skyrmions, which may significantly broaden our capability to design skyrmion‐based devices.

## 5 Experimental Section

### LTEM Measurements

The current‐driven dynamics of skyrmions was explored using the Titan G2 60‐300 (FEI) in the Lorentz TEM mode at an acceleration voltage of 300 kV. The perpendicular magnetic field could be applied to the device by increasing the objective lens.

### Micromagnetic Simulations

The magnetization dynamics were simulated using the standard micromagnetic simulator Object‐Oriented MicroMagnetic Framework (OOMMF).^[^ ^47^ ^]^ The Hamiltonian H is defined as

(1) $$H=-J\sum_{\langle i,j\rangle }m_{i}\cdot m_{j}-K_{u}\sum_{i}{\left(m_{i}^{z}\right)}^{2}-H_{z}\sum_{i}m_{i}^{z}+H_{\mathrm{DDI}}$$

where m_*i*_ represents the normalized magnetization m_*i*_ = *M_i_*/*M*_S_ at the site *i*. *M*_S_ denotes the saturation magnetization. 〈*i*, *j*〉 run the nearest‐neighbor sites. *J* denotes the nearest‐neighbor Heisenberg exchange coupling energy constants. *K*_u_ is the uniaxial anisotropy constant, and *H_z_* is the external magnetic field along the *z*‐axis.

The magnetization dynamics were simulated using the Landau–Lifshitz–Gilbert (LLG) equation augmented with the adiabatic and non‐adiabatic spin‐transfer torques (STTs) provided by electrons that directly flow through the FeSn layer according to the Zhang‐Li model,^[^ ^44^ ^]^ which is expressed as

(2) $$\begin{array}{ccc} \frac{dM}{dt} & = & -\gamma_{0}M\times H_{\mathrm{eff}}+\frac{\alpha }{M_{S}}\left(M\times \frac{dM}{dt}\right)+\frac{u}{M_{S}^{2}}\left(M\times \frac{\partial M}{\partial x}\times M\right)\\ & & -\;\frac{\beta u}{M_{S}}\left(M\times \frac{\partial M}{\partial x}\right) \end{array}$$

where $H_{\mathrm{eff}}=-\left(1/\mu_{0}\right)\cdot \left(\delta H/\delta M\right)$ is the effective field, *μ*_0_ is the vacuum permeability constant, *γ*_0_ is the Gilbert gyromagnetic ratio, and *α* is the phenomenological damping coefficient. The coefficient for Zhang‐Li STT is given as *u* = (|*γ*_0_ℏ/*μ*_0_ *e*|) · (*jP*/2*M*_S_), where ℏ is the reduced Planck constant, *e* is the electron charge, *j* is the applied current density, and *P* is the spin polarization rate. The strength of the non‐adiabatic STT is defined by *β*. As this study only focuses on the magnetization dynamics in the *x*–*y* dimensions, the model was treated as a 2D system. Namely, it was assumed in this work that the spin textures do not vary along the thickness direction. Hence, a mesh size of 5 nm × 5 nm × 5 nm in the *x*, *y*, and *z‐*direction, respectively, was used. The intrinsic magnetic parameters are the saturation magnetization *M*_S_ = 5.66 × 10^5^ A m^−1^, the exchange constants *A* = 1.4 × 10^−11^ J m^−1^, and anisotropy constant *K*_u_ = 0.8 × 10^5^ J m^−3^. The damping coefficient and the non‐adiabatic STT coefficient were set as *α* = *β* = 0.1. Notably, the current‐induced dynamic processes of an elliptical skyrmion were simulated using a much smaller *β* of 0.01 as reported in ref. [48] (see Figure S8, Supporting Information) and the value of *β* affects the switching process slightly. The spin polarization rate is assumed to be *P* = 1.0 for simplicity. The pinning sites were implemented by locally modifying the anisotropy parameter for certain spins. In all simulations, the default stage‐stopping criteria setting of the standard OOMMF simulator was used, that is, a stage should be considered complete when the maximum |dm/dt| across all spins drops below 1° ns^−1^. The pinning region was set by increasing the magnetocrystalline anisotropy at a certain region of the model, as is marked with a white area in the simulation.

## Conflict of Interest

The authors declare no conflict of interest.

## Supporting information

Supporting Information

Supplemental Video 1

Supplemental Video 2

Supplemental Video 3

## Data Availability

The data that support the findings of this study are available from the corresponding author upon reasonable request.
