# Supplementary material for: Current‐Induced Reversible Split of Elliptically Distorted Skyrmions in Geometrically Confined Fe3Sn2 Nanotrack
Source: Adv Sci (Weinh). 2023 Jan 22;10(9):2206106. doi: 10.1002/advs.202206106 (PMC10037979; doi:10.1002/advs.202206106)
Supplement: Supplementary file 1 — Supporting Information [file ADVS-10-2206106-s003.pdf]

Supporting Information

Current-Induced Reversible Split of Elliptically Distorted Skyrmions in Geometrically Confined Fe_3Sn_2 Nanotrack

Zhipeng Hou, Qingping Wang, Qiang Zhang, Senfu Zhang, Chenhui Zhang, Guofu Zhou, Xingsen Gao, Guoping Zhao, Xixiang Zhang*, Wenhong Wang*, Junming Liu*

Keywords: skyrmions, current-driven dynamics, room temperature

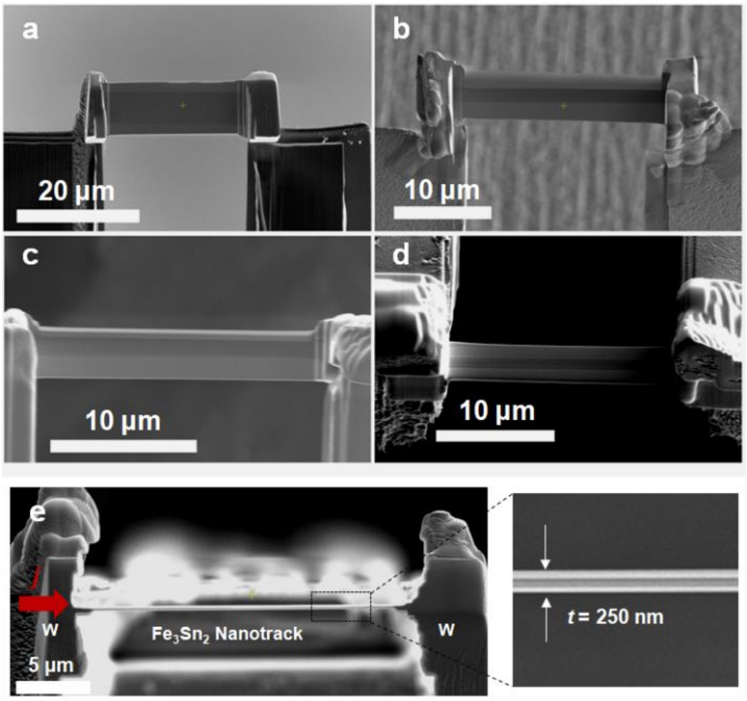

Figure S1 Scanning electron microscopy (SEM) image of the track devices. Top view of the *w* ~ **a** 4.9 µm, **b** 1.4 µm, **c** 1µm, **d** 700 nm track devices. The left panel of **e** shows the side view of the *w* ~ 4.9 µm track device. The thickness of the track is established to be approximately 250 nm.

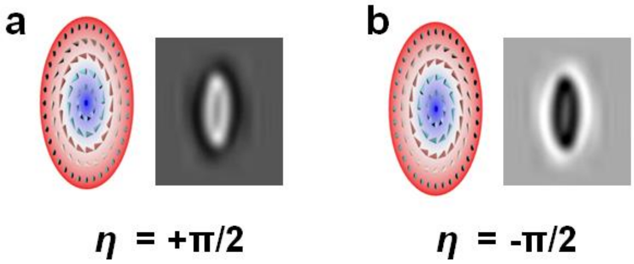

Figure S2 The left panels of **a** and **b** show the schematic view of two skyrmions with opposite helicity, respectively. The right panels of **a** and **b** are the corresponding simulated LTEM images based on their spin texture.

Supplementary Note 1

The Joule heating generated by the current is simulated based on the method given in [*Appl. Phys. Lett.* **2018**, *112*, 212403] by using the MATLAB software. The model size (length l ×width w × thickness t) is 20 μm × 1.4 μm × 250 nm, which is the same as that of the sample in our experiments. The initial temperature is set to be 300 K and the relevant physical parameters for Fe_3Sn_2 are summarized in Table S1. The values of mass density ρ_m, heat capacity C_p and resistivity ρ could be estimated by our experiments [see Fig. S3a and S3b] or previous references. However, the value of thermal conductivity k for Fe_3Sn_2 single crystal is hard to estimate because the Fe_3Sn_2 crystals are usually 1 mm × 0.5 mm hexagons while an 8 mm × 2 mm × 2 mm brick is required for exactly measuring k. Meanwhile, the heat transfer coefficient h_c through the surfaces is also hard to be directly established in experiments. In our simulations, we have referenced the values of k and h_c for the B_{20} compound FeGe [*Appl. Phys. Lett.* **2018**, *112*, 212403]. First, we have simulated the temperature distribution of the nanotrack for k = 3.39 W m^{-1} K^{-1} and h_c = 5×10^6 W m^{-2} K^{-1} (the values of k and h_c for FeGe are referenced) when a current pulse of density j = 3.4 × 10^{10} A m^{-2} and pulse width τ = 100 ns is injected. It is found that the highest temperature (T_h) that the sample could be heated up to is about 480 K (see Fig. S3c). By varying the value of k or h_c, the value of T_h is only slightly affected in such a current density and pulse width (see Fig. S3c). Hence, our simulations are reliable though we use the values of k and h_c for FeGe. We have further simulated the temperature distribution of the nanotrack for j ranging from 3.0 × 10^{10} A/ m^2 to 4.4 × 10^{10} A/m^2. The corresponding value of T_h ranges from 430 K to 690 K (see Fig. S3c). In experiments, we have established that the maximum current density for switching the helicity in the Fe_3Sn_2 nanotrack is established to be approximately 4.2×10^{10} A/m^2, above which the

device could be easily heated beyond the Curie temperature (T_c) of Fe_3Sn_2 (T_c = 640 K). Our simulated results agree with the experiments qualitatively.

Table S1. Parameters for Joule heating simulations

Parameter	Vaule	Units	Notes
ρ_m	8250	kg m^{-3}	*J. Alloys. Comp.* **2006**, *422*, 132
C_p	323	J kg^{-1} K^{-1}	The average value between 400 K and 300 K in Fig. R1a
ρ	329.6(1+0.00354(*T*-300))	μΩ cm	Linear fitting of Fig.R1b from 300 K to 400 K

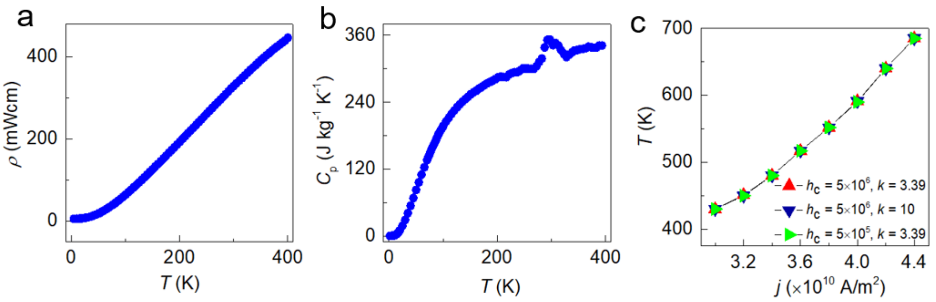

Figure S3 Temperature dependence of **a** ρ and **b** C_p in a temperature range of 10-400 K. **c** Simulated highest temperatures that the sample could be heated up to when parameters are adopted from Table R1. The pulse density ranges from 3.0 × 10^{10} A/ m^2 to 4.4 × 10^{10} A/ m^2 with a fixed pulse width τ of 100 ns. The value of k varies from 3.39 W m^{-1} K^{-1} to 10 W m^{-1} K^{-1} and the values of h_c range from 5×10^5 W m^{-2} K^{-1} to 5×10^6 W m^{-2} K^{-1}. We found that the effect of h and k is insignificant.

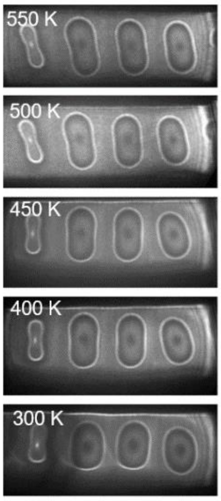

Figure S4 The under-focused LTEM images of the w ~ 1.4 μm nanotrack. The external temperature ranges from 300 K to 550 K with a fixed magnetic field of 140 mT.

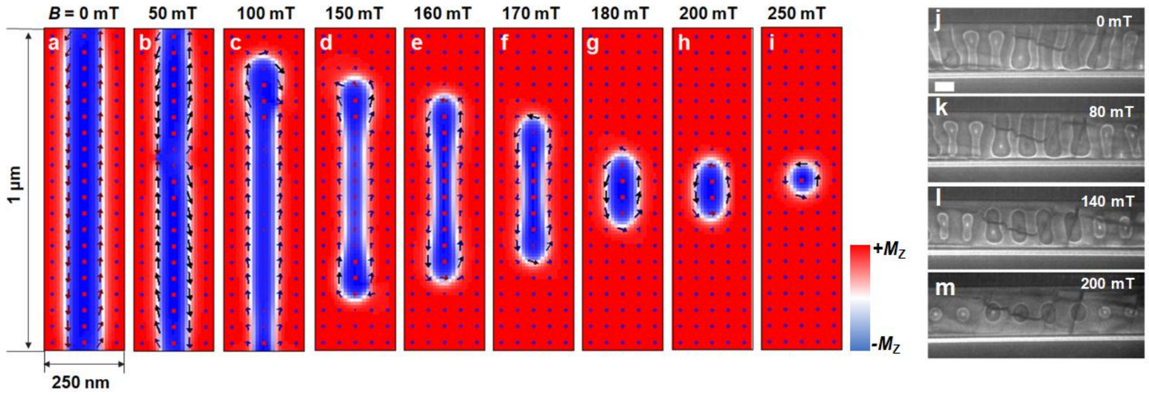

Figure S5 Magnetization dynamics of the elliptical skyrmions. **a-i** The simulated magnetization dynamics of the magnetic domain states in a track with a length of 1 μm, a width of 250 nm, and thickness of 100 nm. The magnetization along the *z*-axis (M_z) is represented by regions in red ($+M_z$) and blue ($-M_z$). **j-m** Experimental magnetization dynamics of the magnetic domain states in a track with a width of 1.4 μm. The scale bar for **j-m** is 500 nm.

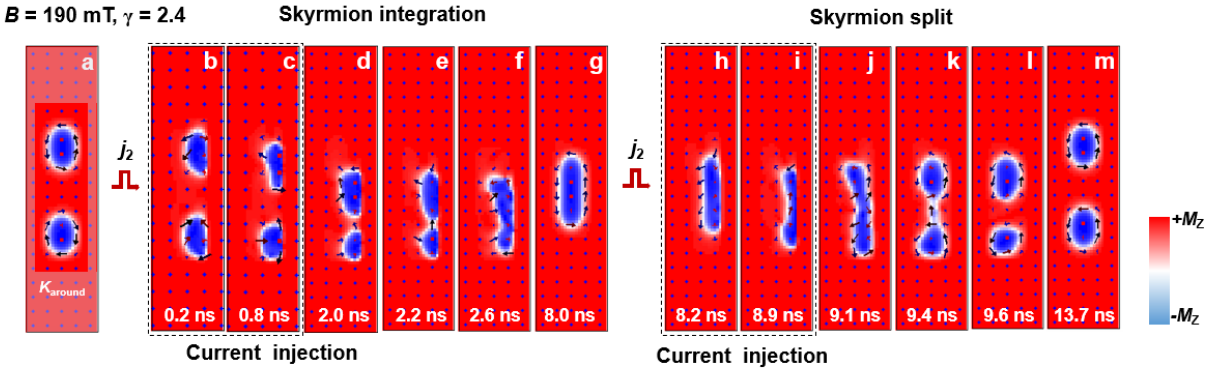

Figure S6 Simulated current-driven dynamics of elliptical skyrmions. **a** Snapshots of an elliptical skyrmion with $\gamma = 2.4$ at $t = 0$ ns (without injection of current). The shadow region represents the pining region with a pinning strength of $K_{\text{around}} = 20K_u$. **b-g** Snapshots at six selected times showing the skyrmion intergration. A spin-polarized current of 3.0×10^{12} A/m^2 is injected during $t = 0 \sim 1$ ns. **h-m** Snapshots at six selected times showing the split of the elliptical skyrmion at a current of 3.0×10^{12} A/m^2. The current is injected during $t = 8.0 \sim 9.0$ ns. An out-of-plane magnetic field (B) of 190 mT is applied for **a-m**. The magnetization along the *z*-axis (M_z) is represented by regions in red ($+M_z$) and blue ($-M_z$).

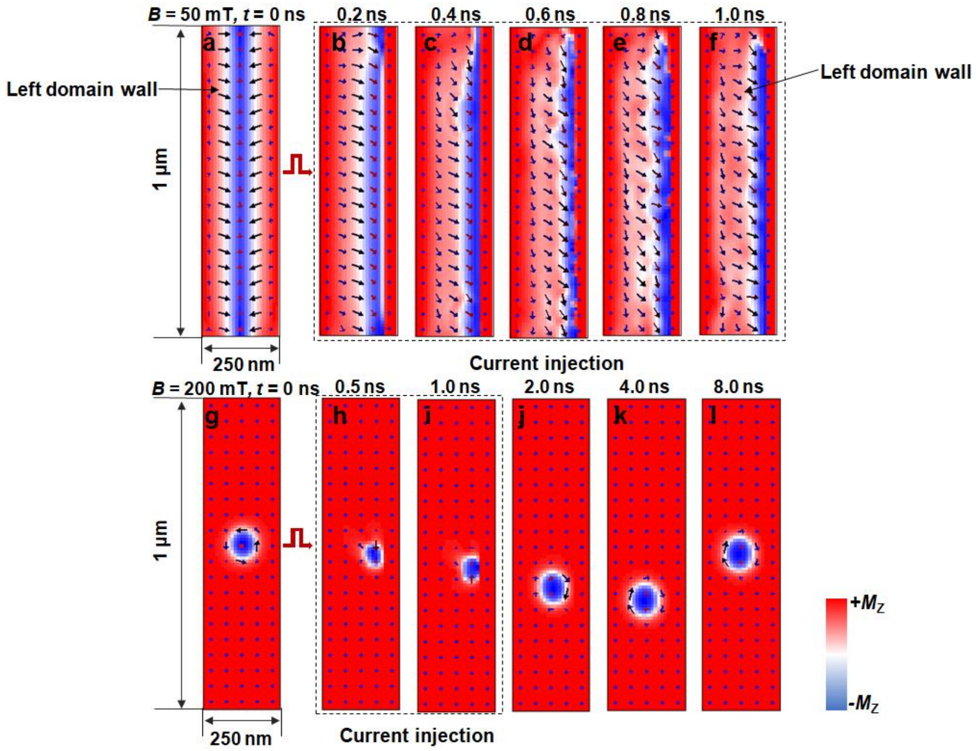

Figure S7 Current-driven dynamics of the stripe domain and circular skyrmion (a circular skyrmion is regarded as a combination of two half skyrmions). **a** Snapshot of a stripe domain under an out-of-plane magnetic field (*B*) of 50 mT at *t* = 0 ns (without injection of current) in a track with a length of 1 μm and width of 250 nm. **b-f** Snapshots at five selected times showing the current-driven dynamics of a stripe domain. A spin-polarized current of 4.0×10^{12} A/m^2 is injected during *t* = 0 ~ 1 ns. We can find that the left domain wall of the stripe domain is significantly squashed with the injection of current. **g** Snapshot of a circular skyrmion under *B* of 200 mT at *t* = 0 ns (without injection of current) in a track with a length of 1 μm and width of 250 nm. **h-l** Snapshots at five selected times showing the current-driven dynamics of a circular skyrmion. A spin-polarized current of 3.0×10^{12} A/m^2 is injected during *t* = 0 ~ 1 ns. We can find that the helicity of the circular skyrmion is reversely switched. The magnetization along the *z*-axis (M_z) is represented by regions in red ($+M_z$) and blue ($-M_z$).

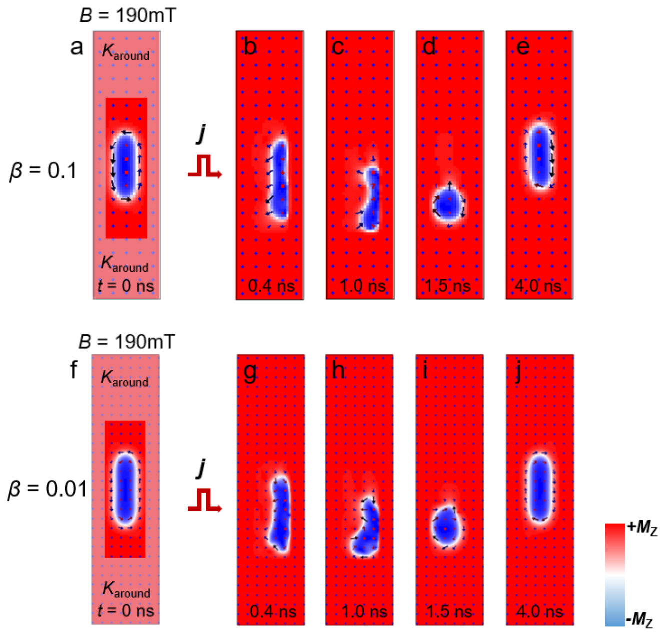

Figure S8 Simulated current-driven dynamics of elliptical skyrmions using different non-adiabatic STT coefficient (β). **a** Snapshots of an elliptical skyrmion at β = 0.1 and t = 0 ns(without injection of current). The length, width, and thickness of the track are 1 μm, 250 nm, and 100 nm, respectively. The shadow region represents the pining region with a pinning strength of $K_{\mathrm{around}} = 20K_{\mathrm{u}}$. **b-e** Snapshots at four selected times showing the helicity switching of the elliptical skyrmion. A spin-polarized current of 2.3 × 10^{12} A/m^2 is injected during t = 0 ~ 1 ns. **f** Snapshots of an elliptical skyrmion at β = 0.01 and t = 0 ns(without injection of current). The length, width, and thickness of the track are 1 μm, 250 nm, and 100 nm, respectively. The shadow region represents the pining region with a pinning strength of $K_{\mathrm{around}} = 20K_{\mathrm{u}}$. **g-j** Snapshots at four selected times showing the helicity switching of the elliptical skyrmion. A spin-polarized current of 2.3 × 10^{12} A/m^2 is injected during t = 0 ~ 1 ns. The value of B for **a-j** is 190 mT. The magnetization along the z-axis (M_z) is represented by regions in red ($+M_z$) and blue ($-M_z$).
